# The Making of a Modern Hospital

**Published:** 1911-11-04

**Authors:** 


### THE MAKING OF A MODERN HOSPITAL.

### IV.—The Departments of a Modern Hospital (*continued*).

Since the managers of a modern up-to-date institution must take into account the recent advances in medicine and surgery which have modified the methods of treatment and the general handling of the sick, it follows that the hospital itself must, if it claims to be up to date in the true sense, be in a position to employ those new methods in the best manner. This fact has considerably increased the number of departments on the medical side of our modern institutions; it has also greatly augmented the cost of upkeep and the percentage cost per bed. Let us see what realisation of the ideal to make the institution the best of its kind implies in modern times.

### The Main Departments.

The two large and principal departments on what may be termed the medical, in contrast to the purely administrative, side are the medical and surgical departments. These in themselves are not solitary, but conglomerations of different "units" or "details," as they are sometimes called. By a "unit" is meant the arrangement whereby the indispensable minimum that modern requirements demand is secured. Thus, we speak of a ward unit, of a mortuary unit, and of a kitchen unit, meaning by these terms the grouping of one or more details into a complete whole, which can be easily and systematically worked. The medical side, and in fact every department, consists of one or more of such units, which, so far as general plan and conception are concerned, may agree very closely in every department, though they may differ widely as to detail. With these units we shall deal at length later on; for the present it is sufficient to mention them and to draw attention to the fact that they are almost indispensable terms of the nomenclature in the discussion of modern hospital problems.

The number of subsidiary departments, apart from the two main ones already referred to—the medical and surgical ones—vary in different hospitals. In some of the older institutions the medical wards receive all cases of disease which are not purely surgical, or, in other words, which do not primarily need operative or manipulative procedure for their treatment. When, through various circumstances, such cases are in need of surgical interference they are either transferred to the surgical wards or are operated on in the medical wards, though here again custom differs widely. As a rule, however, the medical wards are reserved for purely medical cases, and the variety of diseases which may be grouped under one roof is often very startling. Thus in one hospital visited—as indeed in most of our London hospitals—cases of nervous disease, of enteric, of consumption, of gastric disease, and even of mental disease, are to be found almost side by side. The inference is that such cases are not deemed sufficiently "specialised" to warrant their relegation to a special ward exclusively used for one or other class of complaint.

### Extremes of Specialisation.

Newer opinion, however, inclines to specialise, and therefore the modern tendency is to create subsidiary departments on the medical side. The apotheosis of such specialisation has been observed in certain American institutions where we see special departments for nervous diseases, for diseases of the stomach and organs of digestion, for diseases of the brain, for diseases of the heart, and even for diseases of the blood. While sane and conservative hospital opinion deprecates this rather needless multiplication of specialties, it agrees that there are certain subsidiary special departments on the medical side which every modern hospital should possess. These are the isolation department for the reception and treatment of cases of infectious disease; the observation department for the reception of cases unsuitable for admission to the general ward on account of their likelihood to disturb the patients therein; the children's department; the department for nervous diseases, and the skin department. Similarly on the surgical side we have a large general surgical department, with subsidiary departments for the reception and treatment of accidents and casualties, genito-urinary diseases, diseases of women, diseases of the throat, diseases of the nose, diseases of the eye, diseases of the ear, and deformities generally. These are the now generally recognised specialties, but there can be little doubt that as medical science advances further and our means of diagnosis and treatment become more reliable and systematic, it will be found necessary to create other special departments. The reason is that many newer methods of treatment demand for their adequate employment the use of complicated apparatus and appliances which are more conveniently installed in special departments under the control of men who have made a study of these new methods than in the general ward under the supervision of a general surgeon or physician. Another reason is that it is more convenient to treat certain classes of diseases in groups; such an arrangement facilitates treatment and makes the nursing and general direction of the treatment better than if these cases were distributed among the beds in a general ward. Thus, to take one instance, the diseases of the eye demand special conditions in the ward and special facilities for operation, which are more easily secured in a separate room or building than in the general ward; deformities, again, necessitate very highly specialised apparatus, which cannot conveniently be placed and worked in the day room.

### General Subsidiary Departments.

In addition to these distinctive medical and surgical special departments, there are certain other special departments of a modern hospital which are equally indispensable, but which have a range of usefulness embracing both medical and surgical diseases, and which may therefore be said to be common to both sides. Such are the light department, where certain conditions of disease are treated with Finsen, actinic, or Röntgen rays, and to which nowadays usually a small radium department is attached; the *x*-ray department proper, which serves mainly for diagnostic uses, and is not used primarily for treating patients; the serum department for the preparation of vaccines and sera, with which certain cases are treated under special conditions; the pathological department, which, again, is mainly diagnostic; the dispensing department, where medicines are compounded and served out; the photographic department, with the artist's cabinet, where pictorial records of cases are kept; the registrar's department, where full written records of all cases for reference purposes are compiled; the chemical laboratory, where chemical analyses are made; the pathological laboratory, where similar chemical investigations are carried on; the physiological and research department, which serves mainly as an experimental station; the balneo-therapeutic department, where baths and water apparatus are used; the electric department, with the massage department attached; and last, but by no means least, certain "observation departments" included in each unit.

### Co-operation in Activity.

It is, of course, true that in many hospitals some of these departments are combined, but in most of the newer foundations they are kept distinct, and are under entirely separate and independent management, though it must always be remembered that all the departments of a modern hospital are so arranged that they work harmoniously together, the one helping the other, the one serving as a necessary adjunct to the other, and all contributing to enhance the general efficiency of the institution as a whole. Such efficiency is all the more thorough and complete when the surgical side co-operates fully with the medical, as used to be the case at Breslau under the able management of Naunym and Mickulitz, and as is at present the case at Strasburg under the former able clinician. Naunym recognised the fact, so often lost sight of by enthusiastic specialists who can only see the importance of their own specialty, that there are certain overlapping regions—the borderland regions, as he designated them—between medicine and surgery, which can only be effectively explored by the harmonious, co-operant activity of both the surgeon and the physician. In modern institutions this is increasingly recognised to-day, and some of the best results obtained in medicine are due to the realisation of this fact, and the consequent willing co-partnership that it has brought about.

### The Economic Side.

But when we have enumerated these special departments already mentioned, we have not yet finished with the purely medical side of the hospital. There still remain the departments which serve as connecting links, more especially between the medical and administrative side. These are the instrument makers' department, the laboratories, the mortuary, and the "economic" department which comprises the laundry, bakery, and kitchens. All these departments demand for their proper working a full and highly experienced staff of expert workers, who have to be well trained if their activities are to be utilised in the most economic manner. Specialisation has not been accomplished without the creation of specially trained experts, and these again can only be properly educated in their work in special departments, though it remains a truism that the best specialist is he who has a wide range of knowledge and experience and who has a broad purview of the whole of medical science.

In the succeeding articles we will take these departments in detail, starting with the ward unit on the medical side, and show in what manner they have contributed to enhance the efficiency of the modern institution. The mere enumeration, brief as it is, which has been given, will suffice to show the reader the enormous range of activity of the modern hospital. At the same time it will serve, in some measure, to make him conscious of the reasons which have combined to increase the cost of upkeep of the modern hospital. The old institution, with its two departments, was a relatively inexpensive business in comparison with the modern type. When it existed it was easy to keep the cost per bed down to a comparatively small figure. With our rapid progress in treatment and administration, we have enormously increased the usefulness of the hospital, but we have at the same time inevitably and necessarily added to its expensiveness. All these various departments, with their proper equipment, and with the special conditions that they demanded, have necessitated large outlays of capital which is in a measure unproductive so far as a return in money is concerned. These outlays, however, give us a very full return in the decreased mortality, and the increased percentage of cured or relieved patients which the modern hospital turns out. When we come to the consideration of the operating theatre, we shall see that the latest developments in thoracic surgery, for example, have necessitated the building of very expensive and complicated special anæsthetising rooms—the so-called pressure cabinets. This is only one instance of how a special development in one branch of medical science has influenced construction and outlay in one direction, but several others might be added—for instance, serum therapy radium treatment, and modern methods of treatment in genito-urinary diseases, in orthopædics, and in diseases of the throat.

